# Obituary

**Published:** 1875-04

**Authors:** 


Dr. JOSHUA RILEY, of Georgetown, D. C., died recently of angina pectoris, aged 75 years. He graduated at the University of Maryland in 1824, soon after which he commenced the practice of medicine, and located in Georgetown. From 1844 to 1859 he occupied the chair of Materia Medica, in the Medical Department of Columbian College, and for many years was President of the Medical Association of the District of Columbia. At the recent meeting of this Society, appropriate resolutions were passed, and remarks were made by Drs. Grafton Tyler, L. Mackall, Jr., Armistead Peter, Noble Young, J. F. May, and Lieberman.—*N. Y. Medical Record*, March 20, 1875.

We transcribe the above notice from the pages of the *Record*, in order that we may add thereto our tribute of respect to the memory of a noble old man, an honor to his profession and to his kind.

It has been our good fortune to enjoy for many years the acquaintance of Dr. Riley, and, during three or four of these, to listen to his lectures from his Chair of Materia Medica, and always to find in him the judicious physician, the conscientious instructor, the kind and sympathizing friend.

Few men have ever lived lives so blameless as his, and few have gone down to their graves leaving records as stainless. He was the friend of all, the enemy of none. By his sterling integrity of character he won the respect and confidence, and by his kindness and true philanthropy, the love and affection of all who knew him.

He was, in an especial manner, the student's friend ; as instructor, faithful; as counsellor, prompt, sagacious and encouraging.

Two and twenty years, crowded with the changes and vicissitudes of a more than usually eventful life, have passed, since we received his hearty congratulations and kind farewell upon launching our bark upon the broad ocean of professional life, but many more years must glide into the abyss of eternity, and the recollections of his many virtues fade from the "written tablet of the brain," ere we cease to do honor to the memory of a revered preceptor, a well beloved friend. "May he rest in peace!" H.

